# Comparative Transcriptome Analysis of Genes Involved in Anthocyanin Biosynthesis in Red and Green Walnut (*Juglans regia* L.)

**DOI:** 10.3390/molecules23010025

**Published:** 2017-12-22

**Authors:** Yongzhou Li, Xiang Luo, Cuiyun Wu, Shangyin Cao, Yifei Zhou, Bo Jie, Yalong Cao, Haijun Meng, Guoliang Wu

**Affiliations:** 1 College of Horticultural Science, Henan Agricultural University, Zhengzhou 450002, China; yongzhoulee@163.com (Y.L.); 13223716461@163.com (Y.Z.); 15515729635@163.com (B.J.); cyl1994job@163.com (Y.C.); hjmeng@xmu.edu.cn (H.M.); 2 Institute of Fruit Science, China Academy of Agricultural Science, Zhengzhou 450009, China; luoxiang@caas.cn (X.L.); s.y.cao@163.com (S.C.); 3 Xinjiang Production & Construction Corps Key Laboratory of Protection and Utilization of Biological Resources in Tarim Basin, Alar 843300, China; wcyby@163.com; 4 Henan Key Laboratory of fruit and Cucurbit Biology, Zhengzhou 450002, China

**Keywords:** red walnut, leaf and peel, anthocyanins, transcriptome analysis, differentially expressed genes (DEGs), quantitative real-time PCR (qRT-PCR)

## Abstract

Fruit color is an important economic trait. The color of red walnut cultivars is mainly attributed to anthocyanins. The aim of this study was to explore the differences in the molecular mechanism of leaf and peel color change between red and green walnut. A reference transcriptome of walnut was sequenced and annotated to identify genes related to fruit color at the ripening stage. More than 290 million high-quality reads were assembled into 39,411 genes using a combined assembly strategy. Using Illumina digital gene expression profiling, we identified 4568 differentially expressed genes (DEGs) between red and green walnut leaf and 3038 DEGs between red and green walnut peel at the ripening stage. We also identified some transcription factor families (*MYB*, *bHLH*, and *WD40*) involved in the control of anthocyanin biosynthesis. The trends in the expression levels of several genes encoding anthocyanin biosynthetic enzymes and transcription factors in the leaf and peel of red and green walnut were verified by quantitative real-time PCR. Together, our results identified the genes involved in anthocyanin accumulation in red walnut. These data provide a valuable resource for understanding the coloration of red walnut.

## 1. Introduction

Walnut (*Juglans regia* L.) is an economically important fruit tree that is cultivated worldwide. Walnuts are composed of the peel, shell, and kernel. The kernel is very nutritious, and has been referred to as “the greatest super food” [1]. Walnut kernels are rich in unsaturated fatty acids, protein, carbohydrates, cellulose, vitamins, calcium, phosphorus, iron, and a variety of polyphenols with antioxidant properties [2]. Both the leaf and peel of cultivated walnut varieties are green, and the kernel is yellowish-white or brown. Some red-kernel walnut varieties exist in China, and these may be rare and precious resources for walnut breeding [2]. Recently, the new red walnut cultivar ‘Robert Livermore’, which is rich in anthocyanins, was developed in the USA [3]. Red walnut achieves a higher market price than the traditional varieties because of its unique color. 

Anthocyanins are secondary metabolites that play significant roles in pigmentation. Previous studies have shown that anthocyanin accumulation can improve resistance to *Botrytis cinerea* in grape [4] and improve the nutritional status of carob [5]. Anthocyanins participate in defense responses against pathogens, and protect plants from strong light and ultraviolet radiation. They have antioxidant properties and scavenge free radicals produced in cells under stress [6,7,8,9]. Anthocyanins have been shown to have anti-tumor activities [10,11,12,13] and to protect against coronary heart disease. Carotenoids and anthocyanins affect fruit and leaf color, and their accumulation in fruits and vegetables determines maturity and quality. The most widespread anthocyanins in plants are pelargonidin, cyanidin, delphinidin, paeonidin, petunidin, and malvidin [14]. Anthocyanins accumulate in flowers and fruits of plants and are responsible for the rich colors that attract pollinators and seed dispersers [15]. 

In *Arabidopsis thaliana*, the structural enzymes in the anthocyanin biosynthetic pathway include the upstream components chalcone synthase (encoded by *CHS*) and chalcone isomerase (encoded by *CHI*) and the downstream components dihydroflavonol 4-reductase (encoded by *DFR*), anthocyanidin synthase (encoded by *ANS*), and others [16]. The genes encoding these enzymes show different expression patterns in different species during growth [17]. As well as structural genes, transcription factors encoded by *MYB*, basic helix-loop-helix (*bHLH*) and *WD40* also play important roles in controlling the expression of genes in the anthocyanin biosynthetic pathway [18,19,20]. In some cases, three transcription factors aggregate (e.g., *MYB-bHLH-WD40*) to regulate the expression of genes in the anthocyanin biosynthetic pathway [21]. The structural genes and transcription factors involved in anthocyanin biosynthesis have been identified in genetic analyses of model fruit trees such as apple, grape, and peach [22,23,24,25]. To date, the molecular mechanism of pigment accumulation in red walnut has not been studied in detail. There are many studies on anthocyanin synthesis in fruit trees, but we did not find any study of red walnut yet, so this study can enrich the knowledge about the metabolic mechanisms of red walnut.

Previously, we reported the pigment components of red walnut [26], but there have been no other reports on the pigments in red walnut leaf and peel or on the genes involved in their biosynthesis. Transcriptome analyses based on deep sequencing have been widely used for gene discovery, analysis of specific genes, and to estimate overall gene expression patterns in different varieties and/or tissues. With the publication of the complete genome sequence for walnut [27], transcriptomic analysis is an effective method to identify differentially expressed genes (DEGs) between red and green leaves and peel. Transcriptome sequencing of the leaf and peel at the ripening stage of walnut will provide useful insights into the molecular mechanisms of color development in these tissues. In this study, we analyzed the pigment levels in leaf and peel during the ripening stage of walnut. The leaf and peel transcriptomes were compared between green and red walnut to identify DEGs, especially those involved in pigment synthesis. The results of this study will be helpful not only for understanding the molecular mechanism of anthocyanin accumulation in walnut, but also for the development of molecular markers to use in selective breeding programs.

## 2. Results

### 2.1. Anthocyanin Composition and Content

The leaf of red walnut at the ripening stage contained four anthocyanins; delphinidin-3-*O*-galactoside, delphinidin-3-*O*-glucoside, cyanidin-3-*O*-galactoside, and cyanidin 3-*O*-arabinoside (Table 1). The major anthocyanin was cyanidin-3-*O*-galactoside with 0.676 mg kg^−1^. Only delphinidin-3-*O*-glucoside at 0.283 mg kg^−1^ was detected at fruit ripening stage of red walnut peel. However, we don’t find any component of anthocyanin biosynthesis in the leaf and peel of green walnut. 

### 2.2. mRNA Sequence

Twelve cDNA libraries were constructed from total RNA extracted from the leaf and fruit peel of the red and green cultivars (three replicates per tissue; red leaf: RY1, RY2, RY3; green leaf: NY1, NY2, NY3; red peel: RP1, RP2, RP3; green peel: NP1, NP2, and NP3). These cDNA libraries were subjected to pair-end reading with the Illumina Hiseq 2500 platform, and generated 21–33 million paired-end raw reads 100-bp in length (Table 2). After removing low-quality reads and trimming adapter sequences, we obtained 20–32 million clean reads for each library. Three biological replicates were used for RNA-seq. In correlation analyses, the R^2^ of the three replicates for each sample was greater than 0.95, which confirmed the consistency among the three biological replicates. Thus, the RNA-seq results were confirmed to be highly reliable for further analyses.

### 2.3. Sequence Alignment and Mapping to the Reference Genome

We used the public walnut genome (https://www.ncbi.nlm.nih.gov/genome/?term=Juglans%20regia) [24] as the reference genome. After quality control, the majority of the reads from red walnut leaf (82.79–83.77%), green walnut leaf (84.01–84.38%), red walnut peel (85.11–86.16%), and green walnut peel (84.51–85.25%) were successfully aligned to the reference genome. Most of the reads were aligned to a single position. After quality control, the reads from red walnut leaf (76.21–77.26%), green walnut leaf (77.39–77.47%), red walnut peel (77.81–78.87%), and green walnut peel (77.93–78.92%) were successfully aligned to the reference genome. Approximately equal numbers of reads from red and green walnut at the fruit ripening stage mapped to ‘+’ and ‘−’ strands. The number of non-spliced reads was approximately three times that of spliced reads (Table 3).

### 2.4. Analysis of Differentially Expressed Genes

To identify differences in gene expression between the two cultivars at the ripening stage, we normalized the gene expression levels to the Fragments Per Kilobase per Million (FPKM) values. All uniquely mapped reads were used to calculate the genes’ FPKM values. The DEGs were hierarchically clustered based on the log10 FPKM of the 12 samples, which allowed us to observe the overall gene expression patterns. 

**Figure 1:**
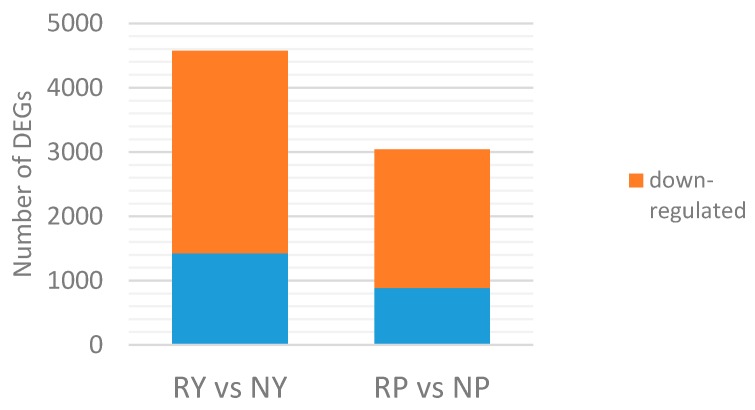
Unigenes differentially expressed between two different cultivars at fruit ripening stage. Blue: up-regulated genes; orange: down-regulated genes.

We used the differential expression gene clustering method to identify DEGs in the leaf and peel between red and green walnut, and constructed a heatmap (Figure S1). The DEGs were identified and filtered using the following criteria: corrected *p*-value < 0.005 and log2 (fold change) value > 1. We compared the DEGs between cultivars within a specific stage. We compared the DEGs in the leaf and peel between cultivars at the fruit ripening stage. The number of DEGs between compared groups ranged from 886 to 3149 (average, 1136) (Table S1). In the leaf, 1423 genes were up-regulated and 3149 genes were down-regulated in red walnut, compared with green walnut. In the peel, 886 genes were up-regulated and 2154 genes were down-regulated in red walnut, compared with green walnut (Figure 1). 

### 2.5. Functional Annotation and Classification

To functionally annotate the red and green walnut transcriptome, 4568 unigenes in the leaf of red and green walnut were blasted against six public databases (Table S2). In total, 1726, 2902, 1384, 4541, 3578, and 4471 unigenes were annotated in the COG, GO, KEGG, NR, Swiss-Prot, and eggNOG database, respectively. Similarly, 3038 unigenes in the peel of red and green walnut were blasted against six public databases (Table S3), and 1152, 1939, 1008, 3014, 2368, and 2969 unigenes were annotated in the COG, GO, KEGG, NR, Swiss-Prot, and eggNOG database, respectively.

**Figure 2:**
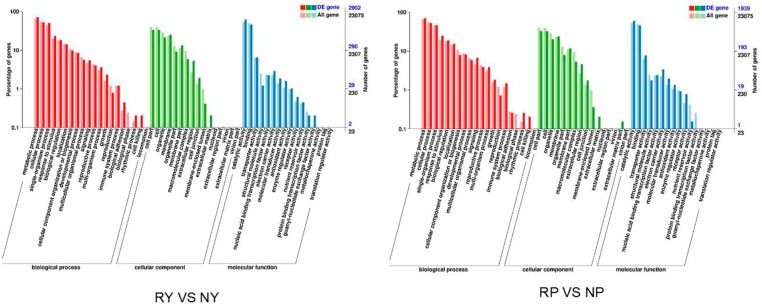
GO classification of unigenes in *Juglans regia* L. leaf and peel. Results are summarized in three main GO categories: biological process, cellular component, and molecular function.

Gene Ontology (GO, http://www.geneontology.org/) is an international standardized system used to classify the function of the predicted genes. There are three major GO categories: molecular function, biological process, and cellular component. The DEGs between red and green walnut leaf and between red and green walnut peel were subjected to a GO enrichment analysis. In total, there were 52 significant terms in the biological process, cellular component, and molecular function categories (Figure 2, Table S4). The anthocyanins that confer the red leaf and peel color of red walnut are synthesized in the endoplasmic reticulum and transported into vacuole by a series of transporters [28]. Anthocyanin-containing compound biosynthetic process (GO:009718), regulation of anthocyanin biosynthetic process (GO:0031540), regulation of anthocyanin metabolic process (GO:0031537), anthocyanin containing compound metabolic process (GO:0046283), and anthocyanin accumulation in tissues in response to UV light (GO:0043481) were identified as subcategories that may contain candidate genes related to anthocyanin synthesis, transport, and metabolism.

To further evaluate the completeness of our transcriptome library and the effectiveness of the annotation process, COG annotations were selected, and 2998 unigenes in the leaf of red and green walnut were assigned to 26 COG categories (Figure S2). Among these categories, general functional prediction (588 unigenes, 19.61%) represented the largest group, followed by translation (378 unigenes, 12.61%), replication, recombination, and repair (352 unigenes, 11.74%) and signal transduction mechanisms (351 unigenes, 11.71%).

The smaller groups were chromatin structure and dynamics (11 unigenes, 0.37%), nucleotide metabolism and transport (19 unigenes, 0.63%), and intracellular trafficking and secretion (16 unigenes, 0.53%). The cell motility, nuclear structure, and extracellular structures groups did not contain any unigenes. In the peel of red and green walnut, 2091 unigenes were assigned to 26 COG categories (Figure S2). Among these categories, general functional prediction (432 unigenes, 20.66%) represented the largest group, followed by signal transduction mechanisms (282 unigenes, 13.49%), translation (278 unigenes, 13.3%) and replication, recombination, and repair (256 unigenes, 12.24%). The smaller groups were chromatin structure and dynamics (four unigenes, 0.19%), nucleotide metabolism and transport (nine unigenes, 0.43%), intracellular trafficking and secretion (9 unigenes, 0.43%), and cell motility (one unigene, 0.03%). The cell motility, nuclear structure, and extracellular structures groups did not contain any unigenes.

**Figure 3:**
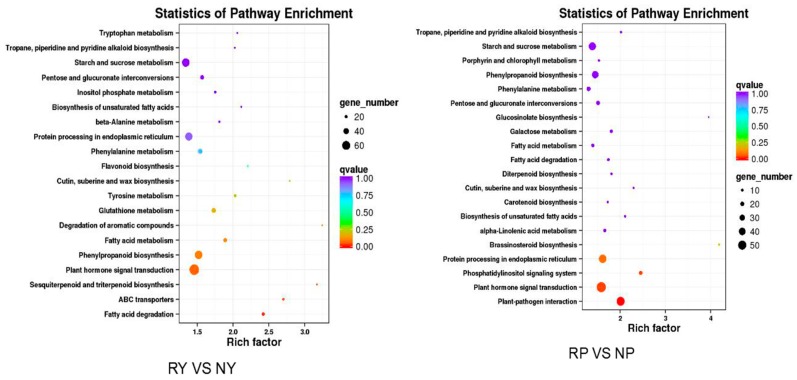
Statistics of KEGG pathway enrichment. Vertical axis shows pathways, horizontal axis shows enrichment factor. Size of dot represents number of DEGs in the pathway. Dot color corresponds to scope of *Q*-value.

To identify the biological pathways activated in the leaf and peel of walnut, pathways enriched with DEGs were identified using tools at the KEGG database [29]. The plant hormone signal transduction pathway was enriched in the leaf of red and green walnut, and the plant-pathogen interaction pathway was enriched in the peel of red and green walnut (Figure 3, Table S5).We found 72 and 57 DEGs related to plant hormone signal transduction in the leaf and peel, respectively, and 18 of them were found in both the leaf and peel. Among these DEGs, 22 were up-regulated in the leaf, and 17 were up-regulated in the peel of red walnut, compared with green walnut. More genes were up-regulated in the leaf than in the peel of red walnut.

Approximately 90% of the reads from red and green walnut at the fruit ripening stage were mapped to exons, 3% to introns, and 4% to 7% to intergenic regions (Figure S3). The reads mapped to introns included those of residual pre-mRNA and introns retained during alternative splicing. Some reads were mapped to intergenic regions as a result of incomplete genome annotation.

### 2.6. Genes from Walnut Transcriptome Involved in Anthocyanin Biosynthesis

Based on annotations in public databases, four DEGs predicted to be involved in flavonoid/anthocyanin biosynthesis were identified in the leaf of red and green walnut (Table 4). *Jrgene13135*, *Jrgene36733*, *Jrgene1355*, and *Jrgene14301* were annotated as *PAL* (encoding phenylalanine ammonia-lyase), *CHS*, *F3′5′H* (encoding flavonoid 3′,5′-hydroxylase), and *UFGT* (encoding UDP glucose: flavonol 3-*O*-glucosyltransferase), respectively. 

Five DEGs in the peel of red and green walnut were predicted to participate in flavonoid/anthocyanin biosynthesis (Table 5): *Jrgene13135*, *Jrgene4994*, *Jrgene30190*, *Jrgene39130*, and *Jrgene39777* were annotated as *PAL*, *CHS*, *F3′5′H*, *F3H* (encoding flavanone 3-hydroxylase), and *UFGT*, respectively.

The spatial and temporal expression of structural genes in the anthocyanin biosynthesis pathway such as *F3H*, *DFR*, and *ANS* are controlled by transcription factors in the *MYB*, *bHLH*, and *WD40* families [30,31]. We found that one *MYB*, one *bHLH*, and one *WD40* (*Jrgene32450*, *Jrgene32411*, and *Jrgene7876*, respectively) were up-regulated in red walnut leaf and peel. The transcript levels of these transcription factors were positively related to the anthocyanin level in the leaf and peel of red walnut.

**Figure 4:**
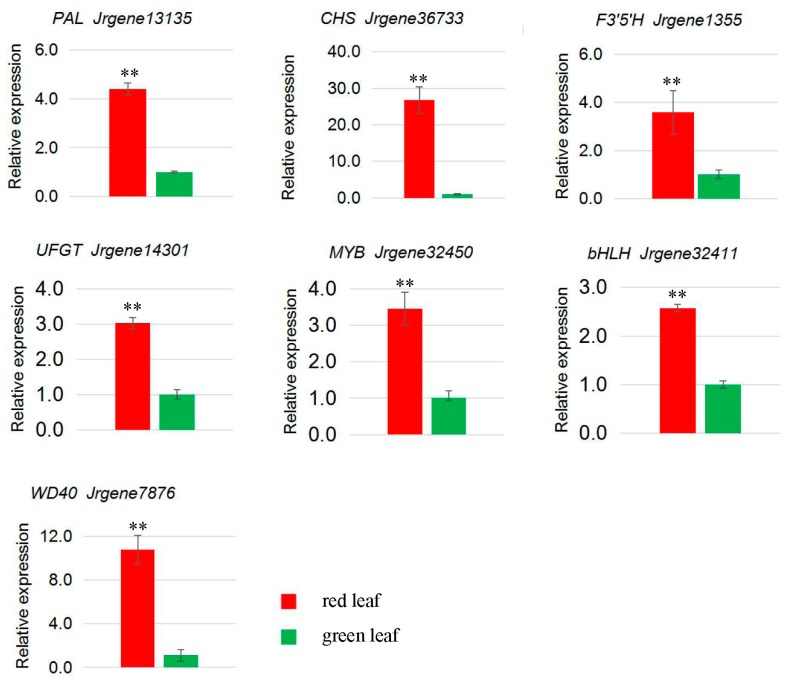
Expression analysis of seven candidate differentially expressed genes related to anthocyanin biosynthesis in leaf of red and green walnut at the fruit ripening stage by qRT-PCR. Relative transcript levels were calculated by ddCt method with *Actin* as the standard. ** *p* < 0.01.

**Figure 5:**
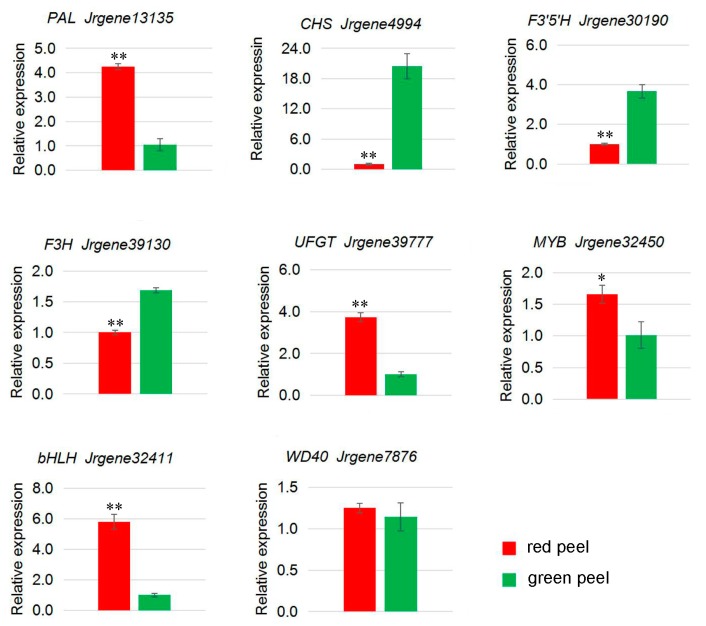
Expression analysis of eight candidate differentially expressed genes related to anthocyanin biosynthesis in the peel of red and green walnut at the fruit ripening stage by qRT-PCR. Relative transcript levels were calculated by the ddCt method with *Actin* as the standard. * *p* < 0.05, ** *p* < 0.01.

To confirm the unigenes identified from the sequencing data and further analyze the differences in gene expression profiles between the red and green leaf and peel at the ripening stage, seven unigenes related to anthocyanin biosynthesis in the leaf of red and green walnut (Figure 4) and eight unigenes in the peel of red and green walnut (Figure 5) were selected for qRT-PCR analyses. The genes showed different transcription patterns and differences in transcript abundance between the leaf and peel in red and green walnut. These qRT-PCR results were consistent with those obtained from the DGE expression profiling. 

## 3. Discussion

The anthocyanins detected in the leaf and peel of red walnut were delphinidin-3-*O*-galactoside, delphinidin-3-*O*-glucoside, cyanidin-3-*O*-galactoside, and cyanidin-3-*O*-arabinoside. The major anthocyanin was cyanidin-3-*O*-galactoside and microcrystalline delphinidin-3-*O*-glucoside was detected in red walnut peel at the fruit ripening stage. The anthocyanins that accumulate in red sweet cherry ‘Tieton’ during the ripening stage are cyanidin-3-*O*-rutinoside and cyanidin-3-*O*-glucoside, and these anthocyanins remain at low levels in yellow sweet cherry ‘13–33’ [32]. In grape, the main anthocyanin is malvidin-3,5-*O*-diglucoside [33]. Thus, the types and quantities of anthocyanins differ among different fruit species. In this study, we detected differences in the composition and concentrations of anthocyanins between the leaf and peel of red walnut. We also quantified differences in pigment accumulation between green and red walnut tissues. The results suggested that the pathways of anthocyanin synthesis differ between the leaf and peel of red walnut, and confirmed that the difference in coloration between red and green walnut is due to anthocyanin accumulation. The phenotypic differences were consistent with the results that the DEGs differed between the leaf and peel of red and green walnut.

**Figure 6:**
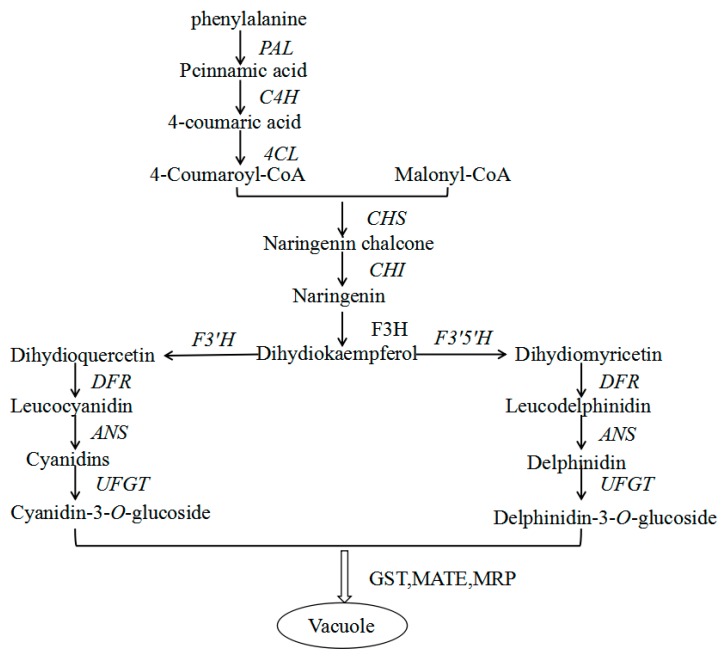
Anthocyanin biosynthetic pathway. Abbreviations: *PAL*, phenylalanine ammonia lyase; *C4H*, cinnamate-4-hydroxylase; *4CL*, 4-coumaroyl-coA synthase; *CHS*, chalcone synthase; *CHI*, chalcone isomerase; *F3′H*, flavonoid-3′-hydroxylase; *F3′5′H*, flavonoid-3′5′-hydroxylase; *F3H*, flavanone-3-hydroxylase; *DFR*, dihydroflavonol 4-reductase; *ANS*, anthocyanidin synthase; *UFGT*, flavonoid-3-O-glucosyltransferase; GST, glutathione S-transferase; MATE, toxic compound extrusion; MRP, multidrug resistance-associated protein.

Anthocyanins are synthesized in the phenylpropanoid and flavonoid pathways in reactions catalyzed by a series of enzymes (Figure 6). Flavonoids are synthesized in the cytosol by biosynthetic enzymes that form a super-molecular complex via protein-protein interactions and anchor in the endoplasmic reticulum membrane [34,35]. The biosynthetic enzymes belong to various families, such as 2-oxoglutarate-dependent dioxygenases (OGD), cytochromes P450 (P450) and glucosyltransferases (GT), suggesting that these enzymes have been recruited for anthocyanin synthesis from pre-existing metabolic pathways [36,37]. In the anthocyanin biosynthetic pathway, PAL catalyzes the first step in the phenylpropanoid pathway. Chalcone synthase, a polyketide synthase, is the first committed enzyme in anthocyanin biosynthesis, and catalyzes the conversion of chalcones to (2*S*)-naringenin, while *CHI* catalyzes the conversion of 2′,4′,4′,6′-tetrahydroxy chalcone to naringenin. The conversion of naringenin to dihydrokaempferol (DHK) is catalyzed by *F3H*, which belongs to the OGD family [38,39]. Flavonoid 3′-hydroxylase (*F3′H*) and flavonoid 3′,5′-hydroxylase (*F3′5′H*) are P450s that catalyze the hydroxylation of DHK to form (2*R*,3*R*)-dihydroquercetin and dihydromyricetin, respectively. They are the key enzymes determining the structures of anthocyanins, and therefore, they affect color formation [19]. Dihydroflavonols are reduced to their corresponding 3,4-*cis*-leucoanthocyanidins by dihydroflavonol 4-reductase (*DFR*). In some plant species such as petunia (*Petunia hybrida*) and cymbidium (*Cymbidium hybrida*), *DFR* has strict substrate specificity and cannot utilize DHK. This is why these species lack anthocyanins and cannot produce flowers with an orange/brick red color. Anthocyanidin synthase (ANS), which belongs to the OGD family, catalyzes the synthesis of corresponding colored anthocyanidins. Anthocyanidins are initially 3-glucosylated by UDP-glucose: flavonoid (or anthocyanidin) 3-glucosyltransferase [40]. The spatial and temporal expression of structural genes in the anthocyanin biosynthetic pathway is determined by a combination of R2R3 *MYB*, *bHLH*, and *WD40*-type transcription factors and their interactions. This has been well established in maize, *Arabidopsis*, petunia, and Japanese morning glory [41,42]. The *WD40* and *bHLH* proteins are pleiotropic, and are involved in multiple processes in addition to anthocyanin synthesis. They are thought to affect these processes via their interactions with specific MYB proteins [39]. In this study, we identified four unigenes related to the anthocyanin pathway in the leaf, and five unigenes related to the anthocyanin pathway in the peel. These unigenes may control color formation in red walnut. In some cases, two or more unigene sequences were annotated with the same gene name. These unigenes may represent different fragments of a single transcript or different members of a gene family [43]. The comparative analysis showed that there were different DEGs encoding the same enzyme in different tissues; i.e., *Jrgene36733* and *Jrgene4994* were annotated as *CHS*, *Jrgene1355* and *Jrgene30190* were annotated as *F3′5′H*, and *Jrgene14301* and *Jrgene39777* were annotated as *UFGT*. We consider that these pairs of unigenes represent homologous genes that function in different tissues. 

Previous studies have shown that anthocyanin content is correlated with the expression levels of certain anthocyanin biosynthetic genes in many fruit crops, such as apple, pear, and grape [22,44,45,46]. In the present study, the RNA-seq data revealed that four anthocyanin structural genes (*PAL*, *CHS*, *F3′5′H*, and *UFGT*) were up-regulated in red walnut leaf compared with green walnut leaf, and these expression patterns were confirmed in the qRT-PCR analyses. In the leaf of red and green walnut at the fruit ripening stage, *Jrgene13135* transcript levels were higher in red walnut than in green walnut. *Jrgene13135* was annotated as *PAL*, which encodes the enzyme that catalyzes the first step of the phenylpropanoid pathway [47]. We did not find any DEGs annotated as *C4H* and *4CL*, which encode the enzymes required for the production of 4-coumaroyl-CoA. The first committed enzyme in the flavonoid pathway is *CHS* [48]. In this study, *Jrgene36733*, annotated as *CHS*, showed a significantly higher expressional level in red walnut than in green walnut. None of the DEGs were annotated as *CHI* or *F3H*. However, the DEG *Jrgene1355*, which was annotated as *F3′5′H*, showed significantly higher expression level in red walnut than in green walnut. Both *F3′H* [49] and *F3′5′H* [50] belong to the cytochrome P450 super family, and catalyze hydroxylation at the 3′ and 3′,5′ positions of the B-ring of the flavonoid to produce the precursors of cyanidin-based anthocyanins and delphinidin-based anthocyanins, respectively. The enzymes encoded by these genes may lead to greater production of flavones and leucodelphinidin, which are the substrates of cyanidin- and delphinidin-based anthocyanins. This is consistent with the fact that cyanidin- and delphinidin-based anthocyanins are the main pigments in red walnut. Dihydroflavonol is reduced by *DFR* to produce anthocyanidin. However, none of the DEGs between the leaf of red and green walnut were annotated as *DFR*. Previous studies have shown that the expression of *UFGT* is critical for fruit coloration in many plants, such as grape, strawberry and lychee [18,19,38,39,51,52]. We found that the transcript level of *UFGT* (*Jrgene14301*) was higher in red walnut leaf than in green walnut leaf. This result indicated that the biosynthesis of anthocyanin compounds is maintained at high levels in the red walnut during the fruit ripening stage. The higher expression levels of *PAL*, *CHS*, *F3′5′H*, and *UFGT* in red walnut leaf than in green walnut leaf suggested that these genes are responsible for color formation in the leaf of red walnut.

*Jrgene13135* was annotated as *PAL*, and its transcript levels were higher in red walnut leaves and peel than in green walnut leaves and peel. There were also significant differences in *CHS* [48] (*Jrgene4994)* transcript levels between red and green walnut, with higher transcript levels in green walnut peel. *Jrgene39130* and *Jrgene30190* were annotated as *F3H* [53] and *F3′5′H* [50], respectively, and both showed significantly higher transcript levels in green walnut peel than in red walnut peel. The opposite pattern was observed in the leaf. The higher transcript levels of these genes in green walnut peel than in red walnut peel is not unexpected, since green tissues can contain abundant colorless flavonoids. In addition, different members of a gene family that encode the same enzyme can be expressed in different tissues, as detected for *CHS*, *F3H*, and *F3′5′H* in this study. In other studies, *CHS* was found to be down-regulated in bicolor dahlia ‘Yuino’ [54], and *F3H* was down-regulated in colored yam [55]. Similarly, we observed that these genes were down-regulated in the peel of red walnut. We detected higher transcript levels of *UFGT* (*Jrgene39777*) in the red walnut peel than in the green walnut peel, suggesting that this gene plays an important role in anthocyanin accumulation in red walnut peel. We did not detect differences in transcript levels of *C4H*, *4CL*, *DFR*, and *CHI* between red and green walnut peel, suggesting that these genes do not affect anthocyanin biosynthesis in red walnut during the fruit ripening stage.

The transcription of genes encoding anthocyanin biosynthesis structural enzymes is controlled by transcription factors in the *MYB*, *bHLH*, and *WD40* families [56,57,58,59,60]. In many species, R2R3 *MYB* transcription factors play important roles in activating the expression of genes involved in anthocyanin biosynthesis. In apple, *MdMYB1/A/10/73* positively regulated anthocyanin synthesis in skin and flesh when co-expressed with *MdbHLH3* or *MdbHLH33* [61,62,63,64,65,66]. In grape, several R2R3 *MYBs* have been implicated in the control of anthocyanin biosynthesis [46]. In strawberry, *FaMYB10* acts as an activator, whereas *FaMYB1* acts as a repressor of anthocyanin biosynthesis, and both are expressed at high levels during the fruit ripening stage [67,68]. Pear *PpMYB10* (previously misassigned as *PyMYB10*) contributes to the red pigmentation of colored pear fruits [69]. In this study, a candidate *MYB* (*Jrgene32450*) showed higher expression in the leaf and peel of red walnut than in the leaf and peel of green walnut during the fruit ripening stage. This gene was identified as a homolog of *Arabidopsis LHY*, which is known to play important roles in anthocyanin biosynthesis [70]. 

A candidate *bHLH* gene (*Jrgene32411*) was also among the DEGs, and showed high homology to *Arabidopsis bHLH75*, which also positively regulates anthocyanin biosynthesis in *Arabidopsis* (https://www.ncbi.nlm.nih.gov/ gene/110224821). Its expression was higher in the leaf and peel of red walnut than in the leaf and peel of green walnut at the fruit ripening stage. A *WD40* (*Jrgene7876*) was up-regulated in the leaf and peel of red walnut compared to green walnut. This unigene showed homology to the *Malus × domestica* gene encoding the *WD40* repeat-containing protein 48 [71]. Its homolog in *Arabidopsis* is known to interact with GL, EGL and PAP to regulate anthocyanin biosynthesis [72]. These results indicate that members of the *MYB*, *bHLH* and *WD40* transcription factor families play key roles in regulating anthocyanin biosynthesis in the leaf and peel of red and green walnut. Many of the DEGs identified in this study are not known to be directly involved in anthocyanin synthesis. Further research is required to analyze the significance of differences in gene expression between red and green walnut.

A previous report introduced and described the phenology of red walnut varieties [3], and we have reported the pigment components of the red variety [28]. Analyses of the physiological characteristics of red walnut leaves (photosynthetic efficiency, fluorescence characteristics) showed that they are similar to those of common walnut. The results of the present study provide a preliminary overview of the molecular mechanism of pigment formation in red walnut. The genes that are up-regulated during color formation in red walnut could be used for gene cloning and marker-assisted selection breeding. In addition, red walnut represents a new cultivar that could be used for hybrid breeding.

## 4. Materials and Methods

### 4.1. Anthocyanin Extraction

The total anthocyanin content in the red walnut cultivars was assayed using the method presented by Kaisu et al. [73,74]. Anthocyanins were extracted in 10 mL of methanol (containing 0.05% hydrochloric acid) for 2 h at 4 °C in darkness. Anthocyanin content was measured at 552 nm.

### 4.2. Total RNA Extraction

The leaf and the peel were collected at the fruit ripening stage, three biological replicates were made at each stage. Total RNA was extracted using a modified CTAB method [75]. RNA degradation and contamination was monitored on 1% agarose gels. RNA purity was checked using a NanoPhotometer^®^ spectrophotometer (Implen, Westlake Village, CA, USA). RNA concentration was measured using a Qubit^®^ RNA Assay Kit in a Qubit^®^ 2.0 Flurometer (Life Technologies, Carlsbad, CA, USA). RNA integrity was assessed using the RNA Nano 6000 Assay Kit of the Agilent Bioanalyzer 2100 system (Agilent Technologies, Santa Clara, CA, USA).

### 4.3. Library Preparation for Transcriptome Sequencing

A total amount of 2 μg RNA per sample was used as input material for the RNA sample preparations. Sequencing libraries were generated using NEBNext UltraTM RNA Library Prep Kit for Illumina (NEB, Ipswich, MA, USA) following manufacturer’s recommendations and index codes were added to attribute sequences to each sample. Briefly, mRNA was purified from total RNA using poly-T oligo-attached magnetic beads. Fragmentation was carried out using divalent cations under elevated temperature in NEBNext First Strand Synthesis Reaction Buffer (5×). First strand cDNA was synthesized using random hexamer primer and M-MuLV Reverse Transcriptase. Second strand cDNA synthesis was subsequently performed using DNA Polymerase I and RNase H. Remaining overhangs were converted into blunt ends via exonuclease/polymerase activities. After adenylation of 3′ ends of DNA fragments, NEBNext Adaptor with hairpin loop structure were ligated to prepare for hybridization. In order to select cDNA fragments of preferentially 240 bp in length, the library fragments were purified with AMPure XP system (Beckman Coulter, Beverly, MA, USA). Then 3 μL USER Enzyme (NEB) was used with size-selected, adaptor-ligated cDNA at 37 °C for 15 min followed by 5 min at 95 °C before PCR. Then PCR was performed with Phusion High-Fidelity DNA polymerase, Universal PCR primers and Index (X) Primer. At last, PCR products were purified (AMPure XP system, Beckman Coulter, Inc., Brea, CA, USA) and library quality was assessed on the Agilent Bioanalyzer 2100 system.

### 4.4. Clustering and Sequencing

The clustering of the index-coded samples was performed on a cBot Cluster Generation System using TruSeq PE Cluster Kit v4-cBot-HS (Illumina) according to the manufacturer’s instructions. After cluster generation, the library preparations were sequenced on an Illumina Hiseq 2500 platform and paired-end reads were generated. 

### 4.5. Transcriptome Assembly

Raw data (raw reads) of fastq format were firstly processed through in-house perl scripts. In this step, clean data(clean reads) were obtained by removing reads containing adapter, reads containing ploy-N and low quality reads from raw data. At the same time, Q20, Q30, GC-content and sequence duplication level of the clean data were calculated. All the downstream analyses were based on clean data with high quality. The adaptor sequences and low-quality sequence reads were removed from the data sets. Raw sequences were transformed into clean reads after data processing. These clean reads were then mapped to the reference genome sequence. Only reads with a perfect match or one mismatch were further analyzed and annotated based on the reference genome. Tophat2 tools soft were used to map with reference genome [76].

### 4.6. Gene Functional Annotation

Gene function was annotated using NCBI BLAST 2.2.28^+^ with an *E*-value threshold of 10^−5^ based on the following databases: NCBI non-redundant protein (Nr) sequences; NCBI non-redundant nucleotide (Nt) sequences; protein family (Pfam) was assigned using the HMMER3.0 package; 12 ukaryotic or Clusters of Orthologous Groups of proteins (KOG/COG); Swiss-Prot (a manually annotated and reviewed protein sequence database); the Kyoto Encyclopedia of Genes and Genomes (KEGG) Ortholog (KO) database categories were assigned to the unigene sequence using the KEGG Automatic Annotation Serve (KAAS) on line [29]; Blast2GO v2.5 (BioBam, Valencia, Spain) was used to obtain Gene Ontology (GO) annotation of unigenes based on BLASTX hits against the Nr database with a cut-off *E*-value of 10^−5^ [77].

### 4.7. Sequence Alignment and Mapping to Reference Transcripts

The Twelve cDNA libraries prepared from the same stage in red and green walnut leaves and peels (RY vs. NY, RP vs. NP) were constructed using the Illumina Truseq RNA Sample preparation Kit. The library preparations were sequenced on the Illumina Hiseq 2500 platform. 

To map the DGE reads, clean reads were obtained by removing low-quality reads and reads containing adapters or poly-N stretches from the raw data. The clean reads were mapped back onto the assembled transcriptome for each sample using RSEM [78].

### 4.8. Differential Gene Expression Analysis

Quantification of gene expression levels Gene expression levels were estimated by fragments per kilobase of transcript per million fragments mapped. The formula is shown as follow: FPKM = cDNA Fragments/[Mapped Fragments (Millions) × Transcript Length (kb)].

For the samples with biological replicates: Differential expression analysis of two groups were performed using the DESeq R package (1.10.1) (R-forge, brussal, Belgium). DESeq provide statistical routines for determining differential expression in digital gene expression data using a model based on the negative binomial distribution. The resulting P values were adjusted using the Benjamini and Hochberg’s approach for controlling the false discovery rate. Genes with an adjusted *p*-value < 0.05 found by DESeq were assigned as differentially expressed.

For the samples without biological replicates: Prior to differential gene expression analysis, for each sequenced library, the read counts were adjusted by edge R program package through one scaling normalized factor. Differential expression analysis of two samples was performed using the DEGseq (2010) R package. *p*-value was adjusted using *q*-value [79]. *q*-value < 0.005 and |log2 (fold change)| ≥ 1 was set as the threshold for significantly differential expression.

GO enrichment analysis of the differentially expressed genes (DEGs) was implemented by the GOseq R packages based Wallenius non-central hyper-geometric distribution [80] which can adjust for gene length bias in DEGs .

KEGG [29] is a database resource for understanding high-level functions and utilities of the biological system, such as the cell, the organism and the ecosystem, from molecular-level information, especially large-scale molecular datasets generated by genome sequencing and other high-throughput experimental technologies (http://www.genome.jp/kegg/). We used KOBAS [81] software to test the statistical enrichment of differential expression genes in KEGG pathways .

### 4.9. qRT-PCR Analysis

Seven unigenes in the leaf of red and green walnut, and eight unigenes in the peel of red and green walnut were chosen for validation using qRT-PCR. Specific primer pairs for selected genes used in qRT-PCR were designed (Table S6).The cDNA was transcribed from 2 μg of total RNA using the PrimeScript^TM^ II 1st Strand cDNA Synthesis Kit in 20 μL of reaction mixture. The qRT-PCR was performed with the LightCyclerR 480 II Detection System (Applied Biosystems, Foster City, CA, USA) with the LightCycler 480 SYBR Green I Master (CWBIO, Beijing, China). The thermal profile for SYBR Green I RT-PCR was 95 °C for 5 min, followed by 45 cycles of 95 °C for 10 s, 60 °C for 10 s and 72 °C for 10 s. Each plate was repeated three times in independent runs for all reference and selected genes. The reference gene (β-ACTIN) was used for normalization. The comparative CT method (2^−ΔΔCT^ method) was used to analyze the expression levels of the different genes [82].

### 4.10. Statistical Analysis

All of the experiments analyzed using data comparisons were repeated three times. Statistical analyses were performed using variance (ANOVA) followed by Duncan’s new multiple range tests with SPSS version 17.0 (SPSS, Chicago, IL, USA). A significance level of *p* < 0.01 was applied.

## 5. Conclusions

Red walnut is a new cultivar with a high anthocyanins content. This study investigated the transcriptome profiles of the leaf and peel in red and green walnut using Illumina RNA-seq technology. The transcriptome analysis identified thousands of DEGs between red and green walnut tissues. We used gene clustering and GO, COG, and KEGG pathway enrichment analyses to describe the transcriptional patterns of genes involved in anthocyanins accumulation. These data provide important information about the molecular mechanism of coloration in red walnut, and will be useful for further research on pigmentation in colored walnut and for the development of molecular markers for breeding.

## Figures and Tables

**Table 1:** Anthocyanin concentrations in red and green walnut tissues at fruit ripening stage.

Period and Position	Anthocyanins (mg kg^−1^)			
	Delphinidin-3-*O*-Galactoside	Delphinidin-3-*O*-Glucoside	Cyanidin-3-*O*-Galactoside	Cyanidin-3-*O*-Arabinoside
Red leaf (RY)	0.080	0.293	0.676	0.123
Green leaf (NY, CK)	ND	ND	ND	ND
Red fruit peel (RP)	ND	0.283	ND	ND
Green fruit peel (NP, CK)	ND	ND	ND	ND

ND stands for not discovered.

**Table 2:** Summary of sequencing data.

Samples Name	Raw Reads	Clean Reads	Clean Bases	GC Content (%)	Q30 (%)
RY-1	23,306,215	22,779,186	6,676,568,814	45.68	94.05
RY-2	24,247,568	23,769,824	6,973,950,086	45.83	94.14
RY-3	21,691,848	21,343,231	6,272,412,100	46.01	93.78
NY-1	23,258,402	22,787,786	6,645,288,228	46.01	93.80
NY-2	26,601,923	26,078,697	7,635,444,098	45.96	93.83
NY-3	24,983,554	24,403,548	7,115,739,742	46.11	93.95
RP-1	24,568,131	24,232,274	7,126,004,890	46.58	93.94
RP-2	33,080,603	32,451,498	9,482,999,428	46.44	94.35
RP-3	28,654,652	28,000,182	8,248,082,522	46.32	93.90
NP-1	22,070,960	21,796,810	6,436,963,652	46.40	93.98
NP-2	21,233,811	20,845,557	6,190,372,176	46.26	93.66
NP-3	22,560,037	21,829,578	6,392,561,574	46.26	94.26

1–3: Three biological replicates of each tissue at fruit ripening stage; Raw reads: original number of reads obtained by sequencing; Clean reads: number of reads after removing low-quality reads and trimming adapter sequences; Clean bases: number of clean reads multiplied by length of clean reads. Q30: Phred score, indicates 99% and 99.9% accuracy of sequenced bases; GC content: percentage of G and C in total bases.

**Table 3:** Clean reads mapped to the reference genome.

Samples Name	Total Reads	Mapped Reads	Uniq Mapped Reads	Multiple Map Reads	Reads Map to ‘+’	Reads Map to ‘−’
RY-1	45,558,372	37,795,360 (82.96%)	34,764,686 (76.31%)	3,030,674 (6.65%)	18,136,253 (39.81%)	18,093,059 (39.71%)
RY-2	47,539,648	39,824,315 (83.77%)	36,730,552 (77.26%)	3,093,763 (6.51%)	19,145,848 (40.27%)	19,100,686 (40.18%)
RY-3	42,686,462	35,339,915 (82.79%)	32,530,754 (76.21%)	2,809,161 (6.58%)	16,959,449 (39.73%)	16,919,275 (39.64%)
NY-1	45,575,572	38,308,495 (84.05%)	35,308,726 (77.47%)	2,999,769 (6.58%)	18,402,601 (40.38%)	18,390,534 (40.35%)
NY-2	52,157,394	43,816,115 (84.01%)	40,369,542 (77.40%)	3,446,573 (6.61%)	21,050,148 (40.36%)	21,040,603 (40.34%)
NY-3	48,807,096	41,183,630 (84.38%)	37,772,027 (77.39%)	3,411,603 (6.99%)	19,718,031 (40.40%)	19,707,282 (40.38%)
RP-1	48,464,548	41,247,795 (85.11%)	37,712,174 (77.81%)	3,535,621 (7.30%)	19,584,181 (40.41%)	19,515,493 (40.27%)
RP-2	64,902,996	55,737,684 (85.88%)	51,160,661 (78.83%)	4,577,023 (7.05%)	26,528,679 (40.87%)	26,456,969 (40.76%)
RP-3	56,000,364	48,249,805 (86.16%)	44,167,278 (78.87%)	4,082,527 (7.29%)	22,926,566 (40.94%)	22,845,563 (40.80%)
NP-1	43,593,620	36,841,808 (84.51%)	33,972,850 (77.93%)	2,868,958 (6.58%)	17,632,332 (40.45%)	17,616,465 (40.41%)
NP-2	41,691,114	35,541,476 (85.25%)	32,900,607 (78.92%)	2,640,869 (6.33%)	17,063,308 (40.93%)	17,047,411 (40.89%)
NP-3	43,659,156	37,161,184 (85.12%)	34,291,341 (78.54%)	2,869,843 (6.57%)	17,795,242 (40.76%)	17,780,682 (40.73%)

**Table 4:** Expression profiles of anthocyanin biosynthesis genes in the leaf of walnut.

Gene_ID	FPKM Value		Annotation
	RY	NY	
*Jrgene13135*	155.58	25.96	*PAL*
*Jrgene36733*	65.03	1.98	*CHS*
*Jrgene1355*	26.26	4.93	*F3′5′H*
*Jrgene14301*	25.70	5.12	*UFGT*
*Jrgene32450*	294.37	156.67	*MYB*
*Jrgene32411*	1.92	0.52	*bHLH*
*Jrgene7876*	81.21	18.15	*WD40*

**Table 5:** Expression profiles of anthocyanin biosynthesis genes in walnut peel.

Gene_ID	FPKM Value		Annotation
	RP	NP	
*Jrgene13135*	286.55	21.74	*PAL*
*Jrgene4994*	0.47	4.70	*CHS*
*Jrgene30190*	0.14	6.09	*F3′5′H*
*Jrgene39130*	0.22	9.03	*F3H*
*Jrgene39777*	52.47	11.28	*UFGT*
*Jrgene32450*	30.76	5.21	*MYB*
*Jrgene32411*	9.50	0.56	*bHLH*
*Jrgene7876*	47.44	31.35	*WD40*

## Supplementary Materials

The following are available online.
