# Supplementary material for: Comparative Transcriptome Analysis of Genes Involved in Anthocyanin Biosynthesis in Red and Green Walnut (Juglans regia L.)
Source: Molecules. 2017 Dec 22;23(1):25. doi: 10.3390/molecules23010025 (PMC5943948; doi:10.3390/molecules23010025)
Supplement: Supplementary file 1 [file molecules-23-00025-s001.zip › Certificate of English Editing.pdf]

Certificate of English Editing

**Date of Issue**	18 December 2017

About the manuscript:

**Title**	Comparative Transcriptome Analysis of Genes Involved in Anthocyanin Biosynthesis in Red and Green Walnut (Juglans regia L.)
**First Author**	Yongzhou Li
**Affiliation**	College of Horticultural Science, Henan Agricultural University, Zhengzhou 450002, China
**Date of editing**	6 December 2017

About the editor:

Editor

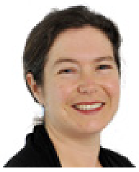

Jennifer Smith

1999 - PhD Botany - University of Otago

Experienced editor, and peer reviewer for Functional Plant Biology and Enzyme and Microbial Technology

Full profile

**Certificate issued by**	Benjamin Shaw<br>Director<br><br>Liwen Bianji (Edanz Group China)

While this certificate confirms the authors have used Edanz's editing services, we cannot guarantee that additional changes have not been made after our edits.

Liwen Bianji (Edanz Group China)
Interchina Commercial Building, 1112A
No 33. Dengshikou Street, Dongcheng District, Beijing, P.C. 100006, China
Phone +86-10-6528-0877 Fax +86-10-6528-0834 Email editing@liwenbianji.cn
