# Supplementary material for: Comparative Transcriptome Analysis of Genes Involved in Anthocyanin Biosynthesis in Red and Green Walnut (Juglans regia L.)
Source: Molecules. 2017 Dec 22;23(1):25. doi: 10.3390/molecules23010025 (PMC5943948; doi:10.3390/molecules23010025)
Supplement: Supplementary file 1 [file molecules-23-00025-s001.zip › S2 Table. Summary of annotations for the assembled walnut in the public databases..pdf]

S2Table. Summary of annotations for the assembled walnut in the public databases.

DEG Set	Total DEGs	COG	GO	KEGG	NR	Swiss-Prot	eggNOG
RY vs NY	4568	1,726	2k,902	1,384	4,541	3,578	4,471
RP vs NP	3038	1,152	1,939	1,008	3,014	2,368	2,969
