# Supplementary material for: Comparative Transcriptome Analysis of Genes Involved in Anthocyanin Biosynthesis in Red and Green Walnut (Juglans regia L.)
Source: Molecules. 2017 Dec 22;23(1):25. doi: 10.3390/molecules23010025 (PMC5943948; doi:10.3390/molecules23010025)
Supplement: Supplementary file 1 [file molecules-23-00025-s001.zip › S6 Table. Primer sequences for qRT-PCR analysis..pdf]

S2a Table. Primer sequences for qRT-PCR analysis in the leaf of red and green walnut.

Genes	Transcripts ID	Forward primer	Reverse primer
PAL	*Jrgene13135*	GCTTTGTTCAGGGATGTG	TGGTGCTTGTAGGTGGG
CHS	*Jrgene36733*	TTATTCATTGGCCCGTAAGC	GGATCTTTGCTGTGGCAAGT
F3'5'H	*Jrgene1355*	GCTCTCGGGTGTTTGTGAAT	TCACCATCCTCTCAGCCATT
UFGT	*Jrgene14301*	AACGAAGACCGAGCCA	AGCCAGGAATGAAGGAAC
MYB	*Jrgene32450*	GGAGAAGGAGGCGGTCATTA	GAATGATGCGCTTCCTGGAG
bHLH	*Jrgene32411*	CAACAAGCAGCTCAGAAAGC	GTAGCTTGGCCTCTTTTTGC
WD40	*Jrgene7876*	CTTGCCAGCACTCCAACATT	CCAGCCAAGAAAGAACCACC
β-ACTIN		GCCGAACGGGAAATTGTC	AGAGATGGCTGGAAGAGG

S2b Table. Primer sequences for qRT-PCR analysis in the peel of red and green walnut.

Genes	Transcripts ID	Forward primer	Reverse primer
PAL	*Jrgene13135*	GCTTTGTTCAGGGATGTG	GGGTGGATGTTCGTGGT
CHS	*Jrgene4994*	ACCCCGATACAAGCGTTGA	AACACCAAATGGGGTGAAAG
F3'5'H	*Jrgene30190*	AGCAATTGATTGGGCACTCT	TCATGAGGAAGCAAAAGTGG
F3H	*Jrgene39130*	CCTTGGTGATCAAATGCAGA	AGCAATCCACAGGTCCAATC
UFGT	*Jrgene39777*	AATACTGCCTGTTGGTCC	TCTTGAGGGCTGTTGC
MYB	*Jrgene32450*	GGAGAAGGAGGCGGTCATTA	GAATGATGCGCTTCCTGGAG
bHLH	*Jrgene32411*	CAACAAGCAGCTCAGAAAGC	GTAGCTTGGCCTCTTTTTGC
WD40	*Jrgene7876*	CTTGCCAGCACTCCAACATT	CCAGCCAAGAAAGAACCACC
β-ACTIN		GCCGAACGGGAAATTGTC	AGAGATGGCTGGAAGAGG
