# Supplementary material for: Comparative Transcriptome Analysis of Genes Involved in Anthocyanin Biosynthesis in Red and Green Walnut (Juglans regia L.)
Source: Molecules. 2017 Dec 22;23(1):25. doi: 10.3390/molecules23010025 (PMC5943948; doi:10.3390/molecules23010025)
Supplement: Supplementary file 1 [file molecules-23-00025-s001.zip › supplemental figure.pdf]

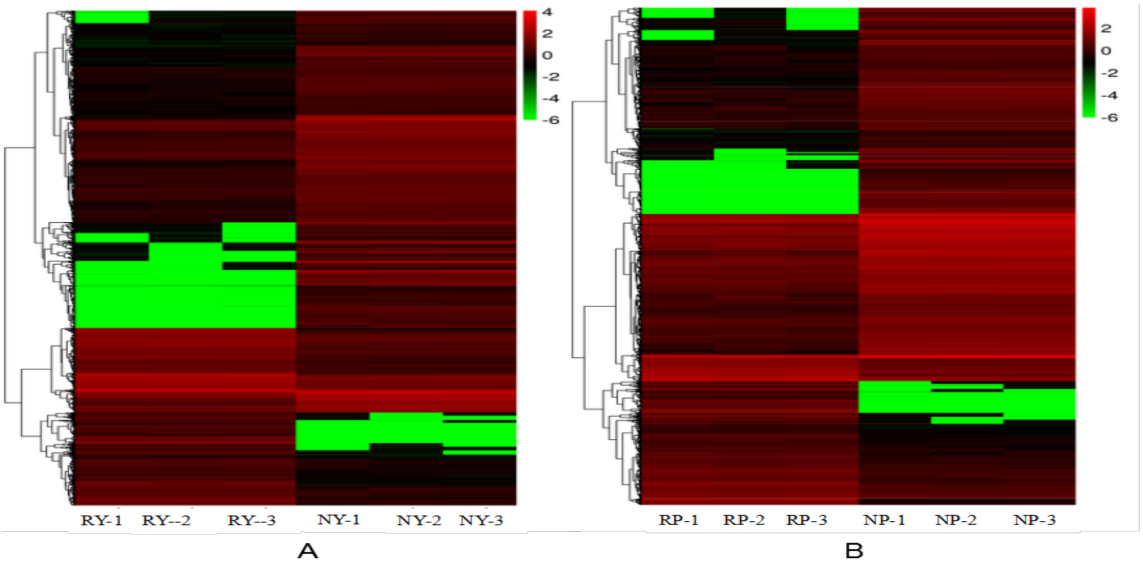


**Fig**.S1 The heatmap of the differential expression genes.A The heatmap of all the differential expression genes in the leaf of red and green walnut.B The heatmap of all the differential expression genes in the peel of red and green walnut.

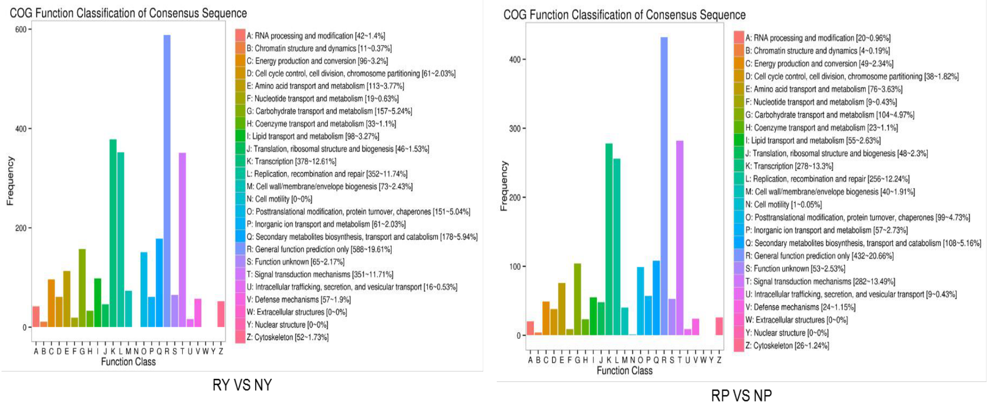


**Fig.S2.**Histogram of COG classification.The leaf of red and green walnut 2998 unigenes and the peel of red and green walnut 2091 unigenes in 26 COG categories.

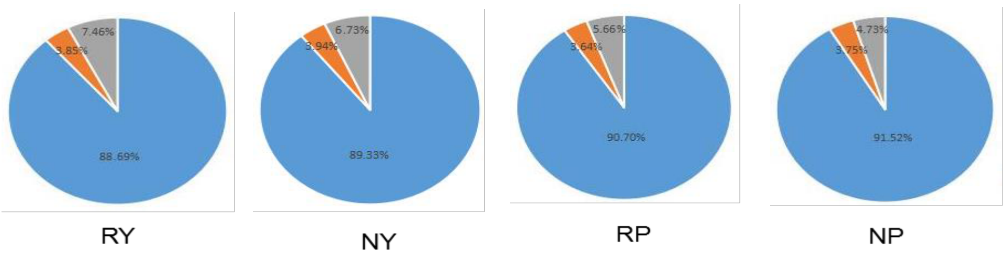


**Fig.**S3 Percent of reads mapped to genome regions. The blue part refers to percentage of reads mapped to exons; the orange part refer to percentage of reads mapped to intergenic regions; the gray part refer to percentage of reads mapped to introns.
